# Post-Disturbance Plant Community Dynamics following a Rare Natural-Origin Fire in a *Tsuga canadensis* Forest

**DOI:** 10.1371/journal.pone.0043867

**Published:** 2012-08-21

**Authors:** Bryan D. Murray, Stacie A. Holmes, Christopher R. Webster, Jill C. Witt

**Affiliations:** 1 Ecosystem Science Center, School of Forest Resources and Environmental Science, Michigan Technological University, Houghton, Michigan, United States of America; 2 U.S. Department of the Interior-Bureau of Indian Affairs, Branch of Forest Resources Planning, Lakewood, Colorado, United States of America; 3 Little River Band of Ottawa Indians, Natural Resources Department, Manistee, Michigan, United States of America; Lakehead University, Canada

## Abstract

Opportunities to directly study infrequent forest disturbance events often lead to valuable information about vegetation dynamics. In mesic temperate forests of North America, stand-replacing crown fire occurs infrequently, with a return interval of 2000–3000 years. Rare chance events, however, may have profound impacts on the developmental trajectories of forest ecosystems. For example, it has been postulated that stand-replacing fire may have been an important factor in the establishment of eastern hemlock (*Tsuga canadensis*) stands in the northern Great Lakes region. Nevertheless, experimental evidence linking hemlock regeneration to non-anthropogenic fire is limited. To clarify this potential relationship, we monitored vegetation dynamics following a rare lightning-origin crown fire in a Wisconsin hemlock-hardwood forest. We also studied vegetation in bulldozer-created fire breaks and adjacent undisturbed forest. Our results indicate that hemlock establishment was rare in the burned area but moderately common in the scarified bulldozer lines compared to the reference area. Early-successional, non-arboreal species including *Rubus* spp., *Vaccinium angustifolium*, sedges (*Carex* spp.), grasses, *Epilobium ciliatum*, and *Pteridium aquilinium* were the most abundant post-fire species. Collectively, our results suggest that competing vegetation and moisture stress resulting from drought may reduce the efficacy of scarification treatments as well as the usefulness of fire for preparing a suitable seedbed for hemlock. The increasing prevalence of growing-season drought suggests that silvicultural strategies based on historic disturbance regimes may need to be reevaluated for mesic species.

## Introduction

Infrequent disturbance events can have a profound effect on forest structure and composition [1]–[3], but they are difficult to study due to their rarity. In mesic temperate forests, fire return intervals can range from several centuries to millennia [2]. Much of our understanding of fire ecology and succession in these forests comes from paleoecological and modeling studies (e.g. [4]–[6]), or the very limited application of prescribed fire (e.g. [7]). Opportunities to directly study post-fire regeneration in unmanaged mesic forest are rare.

The disturbance regime of hemlock-hardwood forests ranges from single-treefall canopy gaps [8]–[10] to intermediate-severity windstorms [11]. Stand-replacing crown fires are infrequent events, with a return interval of 2000–3000 years [2]. However, even age distributions are common in many eastern hemlock (*Tsuga canadensis*) stands, suggesting a stand-leveling disturbance origin [12]–[14]. This observation has led several researchers to conclude that the episodic nature of hemlock regeneration is due in part to hemlock's response to infrequent fires, although the evidence is largely anecdotal [14]–[18]. In contrast, Ziegler et al. [19] reported that burned hemlock forests in Adirondack Park, New York were dominated by deciduous species 90 years after the fire, although the fires occurred in association with logging. The few studies that are available on this topic have typically examined the effects of fire several decades or centuries after the event. Consequently, our understanding of post-catastrophic disturbance vegetation dynamics in this system is limited, especially with regard to the initial interplay between the resurgent plant community, microsite characteristics, and climate. Given changes in regional species pools, legacy effects, and the increasing role of climate change, it is becoming increasingly important to reevaluate these dynamics in contemporary ecosystems, especially for declining “foundation species” (sensu [20]).

Ellison et al. [20] characterize eastern hemlock as a “foundation species” in northern temperate forests of North America. They define a foundation species as “a single species that defines much of the structure of a community by creating locally stable conditions for other species, and by modulating and stabilizing fundamental ecosystem processes” [20], [21]. In the northern Lake States, the paucity of hemlock regeneration has been a concern to ecologists, foresters, and wildlife managers for several decades (e.g. [14], [22]–[28]). Hemlock is important ecologically because it moderates temperature, moisture, and nutrient availability by forming a dense canopy and producing nutrient-poor litter [20]. Maintaining hemlock is important to conserving forest biodiversity because of its close association with several bird [29], terrestrial vertebrate [30], and stream-dwelling [31] species. The structural characteristics of hemlock forests create suitable habitat for white-tailed deer (*Odocoileus virginianus*), birds, mammalian carnivores, and small mammals [32]. Hemlock was an abundant canopy dominant in the region prior to settlement, but is currently found on less than 0.5% of its historic range [26]. Demographic models of existing hemlock-hardwood stands in the Lake States predict that hemlock will lose its status as a canopy dominant over the next several centuries [26], [33]. In the eastern US, hemlock is also threatened by the hemlock woolly adelgid (*Adelges tsugae*) [34].

Low rates of hemlock regeneration in the Lake States have been attributed to several interacting factors including climate change, altered disturbance regimes, hemlock life history traits, ecosystem processes, and past land use practices [26]. Other authors have suggested that a regional increase in white-tailed deer densities during the 20^th^ century is limiting hemlock seedling recruitment into larger size classes [22]–[24], [27]. Rooney et al. [27] demonstrated that seedling establishment was patchy due to local-scale variation in suitable establishment substrates, but that deer browsing is affecting seedling recruitment at the regional scale. Mladenoff and Stearns [26] hypothesized that widespread hemlock recruitment leading to an even-age stand would require an infrequent, catastrophic disturbance followed by several decades of favorably moist climate conditions.

The herbaceous layer (herbaceous and woody plants <50 cm height) is an important component of hemlock forests because it functions as a filter for overstory regeneration [35] and it contains a large proportion of forest biodiversity [36]. Following a stand-replacing disturbance, late-successional species like hemlock, which are shade-tolerant and slow-growing, are often precluded from establishment by fast-growing herbaceous species capable of responding quickly to increased light availability. Alternatively, a disturbance which does not impact the forest overstory may increase regeneration of late-successional species by reducing herbaceous competition and exposing mineral soil seedbeds [37].

We used a serendipitous experiment where a lightning-origin, stand-replacing crown fire burned a section of hemlock-hardwood forest in north-central Wisconsin. As a result of suppression efforts a large portion of the stand remained unburned. Lines of mechanically scarified soil were created in the unburned area when firefighters used a bulldozer to establish fire breaks. The adjacent burned, scarified, and undisturbed sites created an opportunity to compare understory regeneration response following a disturbance that removed much of the canopy and understory layers (fire), a disturbance where the organic soil and understory vegetation was removed without greatly increasing under-canopy solar radiation (analogous to scarification), and no disturbance. Our first aim was to compare the regeneration response of hemlock-hardwood tree and shrub species among the two disturbance types and the undisturbed area, with an emphasis on eastern hemlock regeneration. Second, we examined patterns in herbaceous-layer regeneration in each disturbed area over the first three years after disturbance, while identifying differences in environmental conditions that are influential to regeneration dynamics. We hypothesized that post-fire conditions would favor ruderal herbaceous and shrub species, whereas post-scarification conditions would facilitate the regeneration of late-successional tree species.

## Methods

### Ethics statement

All necessary permits were obtained for the described field studies. Specifically, a permit was issued by the Medford/Park Falls Ranger District, Chequamegon-Nicolet National Forest, United Stated Department of Agriculture Forest Service, Park Falls, Wisconsin, USA.

### Study area

The study area was located in a second-growth hemlock-northern hardwood forest in the Chequamegon National Forest of northwest Wisconsin, USA (45°58′N, 90°00′W). This area receives average annual precipitation of 77.9 cm with mean monthly temperature ranging from −12.2°C in January to 20.1°C in July [38]. The topography of the study area was moderately variable with slopes ranging from 0° to 16°. Overstory vegetation was primarily composed of eastern hemlock with lesser amounts of red maple (*Acer rubrum*), sugar maple (*Acer saccharum*), paper birch (*Betula papyrifera*), balsam fir (*Abies balsamea*), yellow birch (*Betula alleghaniensis*), and hop-hornbeam (*Ostrya virginiana*). Other overstory species noted in the vicinity of the study area included white pine (*Pinus strobus*), red pine (*Pinus resinosa*), northern red oak (*Quercus rubra*), and northern white-cedar (*Thuja occidentalis*). The undisturbed sapling layer (<10 cm DBH and ≥50 cm height) consisted of beaked hazel (*Corylus cornuta*), red maple, eastern hemlock, yellow birch, sugar maple, and balsam fir. The understory layer in the undisturbed area was sparsely vegetated, and characterized by sugar maple seedlings, sedges (*Carex* spp.), and *Dryopteris* ferns.

**Figure 1:**
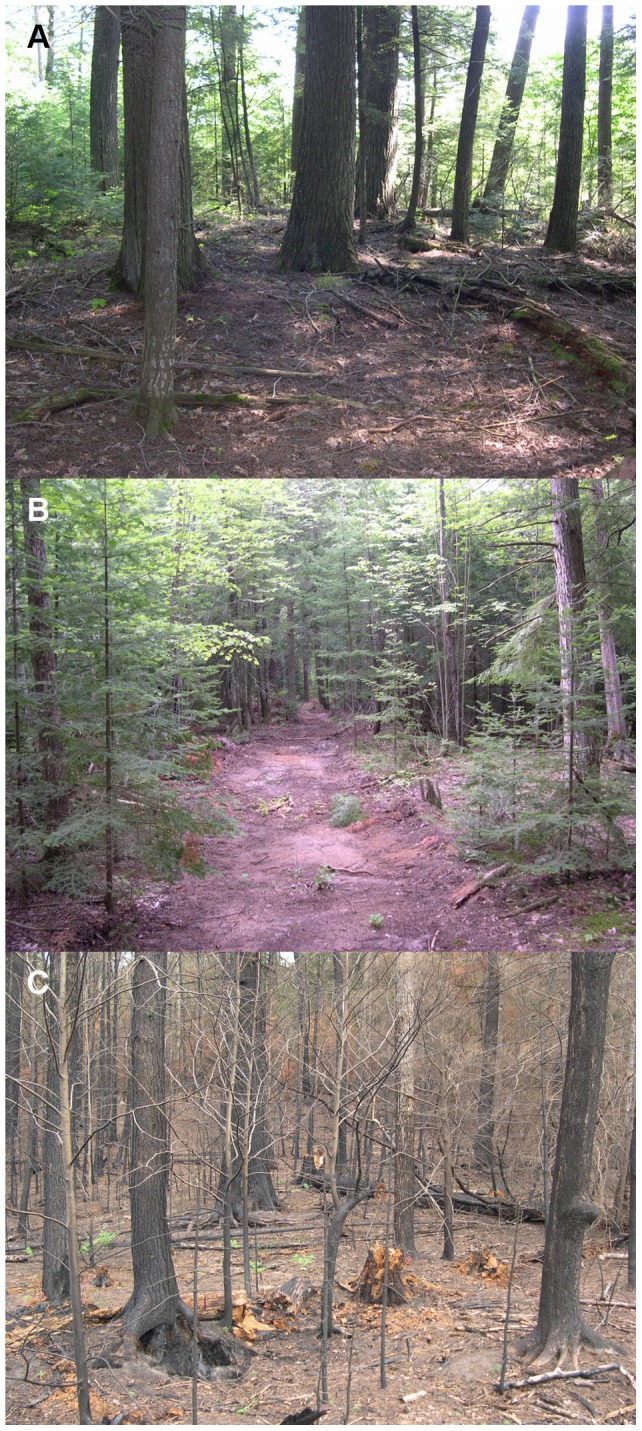
Fire and soil scarification effects in hemlock-hardwood forest. (**a**) Undisturbed hemlock forest was used as a reference site. (**b**) Soil was scarified by a bulldozer used for fire control. (**c**) Portion of hemlock forest was burned by a natural-origin fire in late May 2006.

**Figure 2:**
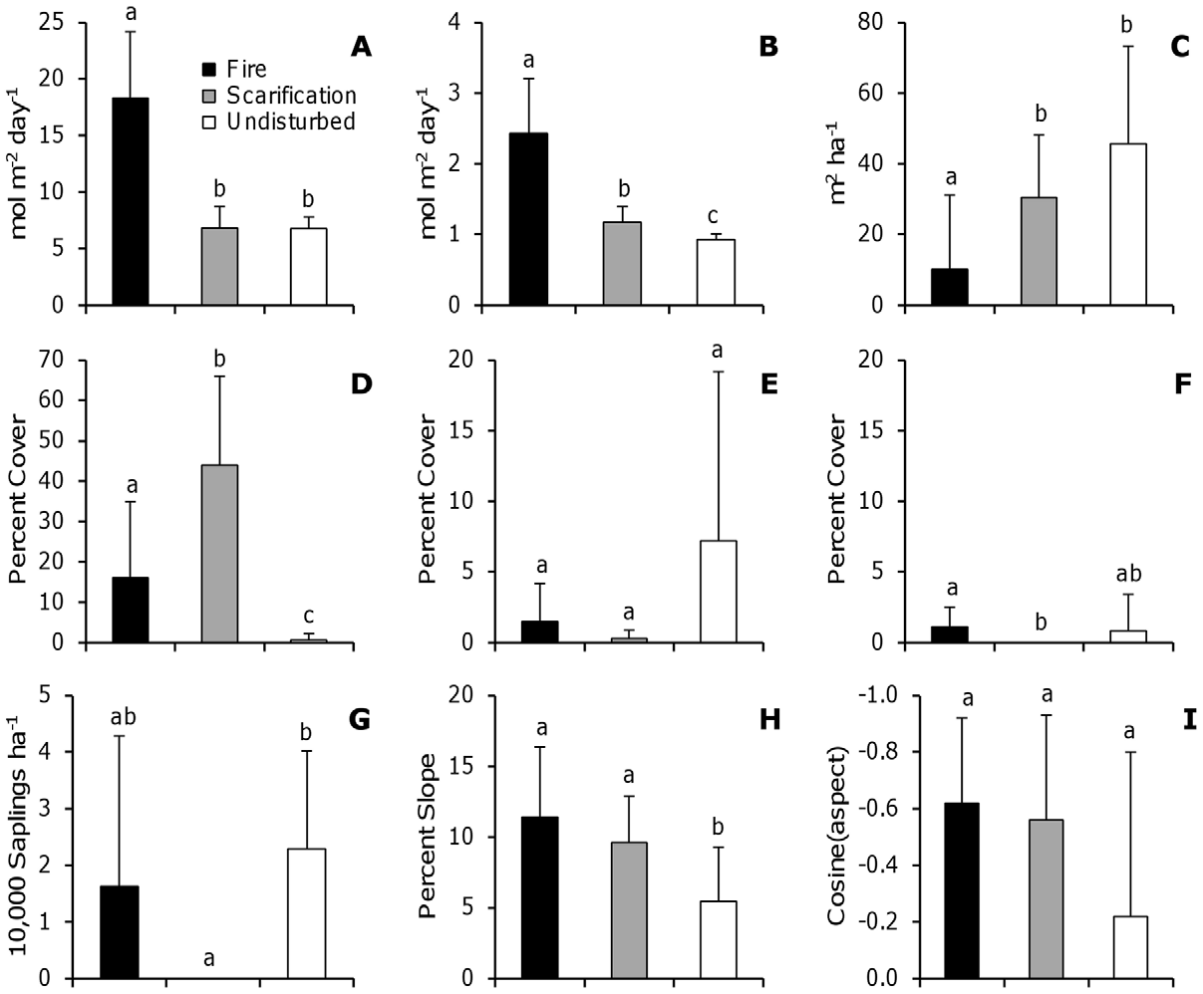
Effect of fire and scarification on the understory environment. Environmental variables (mean ±1 standard deviation; *n* = 10) that were potentially impacted by disturbance: (**a**) direct under-canopy radiation, (**b**) diffuse under-canopy radiation, (**c**) basal area, (**d**) exposed mineral soil, (**e**) low decay coarse woody debris (decay classes 1–3), (**f**) high decay coarse woody debris (decay classes 4 and 5), and (**g**) sapling density. Environmental variables that can affect plant communities: (**h**) slope and (**i**) aspect. Lowercase letters denote results of Mann-Whitney pairwise comparisons by disturbance type, with different letters indicating significant differences at *α* = 0.05. All environmental variables were measured in July 2007, approximately one year after disturbance.

In late May 2006 a lightning strike to a snag initiated a moderate-severity fire at the study site. The fire burned 28 ha of wetlands and mesic northern hardwood forest, including 4 ha of hemlock-dominated forest (S. Adams and M. Lucas, U.S.D.A. Forest Service, personal communication). A bulldozer was used to create a fire break by clearing vegetation (primarily subcanopy trees and herbaceous vegetation) and organic soil in a line through the forest. Much of the hemlock forest remained unburned. This situation allowed us to compare post-disturbance vegetation communities following fire and scarification with those in an undisturbed reference area (Fig. 1). The fire was hot enough to kill herbaceous-layer vegetation, saplings, and overstory trees. It affected stand structure, with a lower live basal area in the burned area (10.3±21.0; mean ±s^2^, n = 10) than in the undisturbed reference area (45.7±27.5). The bulldozer exposed the mineral soil, displaced coarse woody debris, and removed herbaceous- and sapling-layer vegetation. Basal area in the dozer line plots was intermediate between the fire and undisturbed areas (30.4±17.7), suggesting that bulldozer operators may have removed some canopy trees and/or avoided dense patches of larger trees. However, the under-canopy light environment was similar in dozer lines and undisturbed plots, whereas it was significantly greater in the burned area (Fig. 2). The most recent timber harvest at the study site occurred approximately 30 years ago. Evidence of widespread white-tailed deer herbivory was not observed.

### Field methods

We used rebar to permanently mark 10 circular plots of 100 m^2^ area in each of the fire, scarification, and undisturbed areas for a total of 30 plots. Plot locations in the burned and undisturbed areas were selected randomly using ArcView 3.1 Random Point Generator extension (Jenness Enterprises, Flagstaff, AZ, USA) prior to field work. Plots were randomly placed along the center of the dozer line at a minimum of 30 m separation distance. In each plot we measured the diameter of overstory trees ≥10 cm diameter at breast height (DBH; 1.37 m). To measure sapling density, three 3.14 m^2^ circular subplots were nested within each 100 m^2^ plot. In the burned and unburned areas, subplots were placed 3.5 m from the plot center at azimuths of 0°, 120°, and 240°. Dozer line subplots were centered on the dozer line 1 m, 3.5 m, and 4.5 m from the plot center, with the 3.5 m plot opposite the others. The percent cover of herbaceous-layer species, including woody seedlings, was measured in a 1 m^2^ quadrant placed within each subplot. We also measured seedling density by species in the 1 m^2^ quadrants. We defined saplings and seedlings as woody stems <10 cm DBH but ≥50 cm in height, and <50 cm in height, respectively. Plants were identified to species with the exception of sedges, grasses, and mosses. Botanical nomenclature followed Kartesz [39]. Authorities and common names are provided in Table S1. In the 1 m^2^ quadrants, we measured percent cover of exposed mineral soil and coarse woody debris. The decay stage of coarse woody debris segments was ranked on a scale from 1 (no decay) to 5 (highly decayed) according to Jenkins et al. [40]. Light availability was measured in each 1 m^2^ herbaceous-layer plot by taking a hemispheric fisheye photograph at 1.37 m height directly above the quadrant center. We computed diffuse and direct under canopy radiation (mean direct and diffuse photosynthetically active flux density; mol m^−2^ day^−1^) from the fisheye photographs using the computer software WinSCANOPY [41]. Stand structure and light environment were measured one year after disturbance (July 2007), while understory vegetation was sampled for three consecutive years (July 2007, 2008, and 2009).

### Data Analyses

Kruskal-Wallis tests were used to examine differences in environmental variables between disturbance types and the undisturbed area one year after disturbance (July 2007). Environmental variables included aspect (after cosine transformation), overstory basal area, coarse woody debris decay classes 1–3 (low decay coarse woody debris) and 4–5 (high decay coarse woody debris), diffuse under-canopy radiation, direct under-canopy radiation, exposed mineral soil, percent slope, and sapling density. If a significant difference was found (*p*<0.05) then we conducted post-hoc pairwise comparisons using Mann-Whitney tests, where significance was determined using Bonferroni-adjusted *p*-values. We used nonparametric statistical tests because most of the environmental variables did not meet the assumption of normality after Box-Cox transformation. Kruskal-Wallis and Mann-Whitney tests were also used to examine differences in seedling density between disturbance types as well as adjacent years. Statistical tests were conducted in Minitab Release 16 [42].

We used a nonmetric multidimensional scaling ordination (NMDS) to examine complex patterns in herbaceous-layer vegetation [43]. NMDS is a non-parametric method that is useful for examining patterns in multivariate plant community data [43], [44]. We conducted the NMDS using Sørensen's (Bray-Curtis) distance measure in autopilot mode in PC-ORD 5 [45]. Within each 100 m^2^ plot, we calculated the mean percent cover of each species between the three 1 m^2^ quadrants in each year. Percent cover data were arcsine square root-transformed to reduce the stress and improve the interpretability of the NMDS ordination [43]. Rare species with a frequency of <5% were excluded from the analysis. The arcsine square root transformation and removal of rare species reduced the stress of the best solution from 25.0 to 14.2, which is an improvement from an unacceptable to acceptable level of stress [44]. Kendall's *τ* rank correlation coefficient was used to examine relationships between each axis and each species as well as the environmental variables listed previously.

A Multiple Response Permutation Procedure (MRPP) was used to test for differences in species composition between disturbance types. MRPP compares within-group to between-group distances between sample units [43], [46]. The analysis is carried out by first computing a distance matrix, and then an effect size (chance-corrected within-group agreement, *A*) that describes the observed within-group homogeneity compared to expected homogeneity due to chance. Values of *A* range from 1 when there is complete agreement within groups, to 0 when within-group heterogeneity equals random expectation, and can be less than 0 if within-group agreement is less than random expectation. We used Sørensen's distance measure to maintain consistency with the NMDS. We carried out three separate MRPP analyses to test for compositional changes between disturbance types each year after disturbance. If a difference was found, we conducted an indicator species analysis (ISA) [47] to determine which herbaceous-layer species were more prevalent in a given disturbance type. In ISA, an indicator value (*IV*) is computed for each species in each disturbance type by multiplying relative abundance by relative frequency and multiplying by 100. To determine if a species' maximum *IV* was significant, we conducted a Monte Carlo test of significance with 10,000 randomizations [43]. We used an alpha level of 0.05 and minimum *IV* of 25 to determine significance [47]. We used PC-ORD 5 [45] to conduct all multivariate statistical analyses.

We examined differences in the rates of species change over time by computing directional vectors between plot-level NMDS ordination scores from each year of the study. Vectors were standardized by subtracting the tail score from both the head and tail scores, which shifts the tails of all vectors to the plot origin and allows direct comparison between head scores [43]. We used MRPP with Bonferroni-adjusted pairwise comparisons to test for differences in species compositional change between disturbance types from one to two and two to three years after disturbance. Euclidean distance was used because NMDS scores can be less than 0.

## Results

### Environmental Variables

The fire affected both the overstory and ground-layer environment, while dozer scarification primarily affected environmental variables on the ground (Fig. 2). Direct under-canopy radiation was greater in the burned area than either the scarified or undisturbed areas (*H* = 19.05; *df* = 2; *p*<0.001; Fig. 2A). Diffuse under-canopy radiation was greatest in the burned area, intermediate in the scarified area, and lowest in the undisturbed area (*H* = 24.29; *df* = 2; *p*<0.001; Fig. 2B). Live basal area was greater in the undisturbed and scarified areas than in the burned area (*H* = 11.68; *df* = 2; *p* = 0.003; Fig. 2C). Soil scarification resulted in more exposed mineral soil than burning (*H* = 18.50; *df* = 2; *p*<0.001; Fig. 2D). Percent cover of low decay coarse woody debris did not differ by disturbance type (*H* = 1.34; *df* = 2; *p* = 0.512), although variance appeared to be greater in the undisturbed area (Fig. 2E). Percent cover of high decay coarse woody debris was greater in the burned area compared to the scarified area, and was intermediate in the undisturbed area (*H* = 7.57; *df* = 2; *p* = 0.023; Fig. 2F). High decay coarse woody debris was absent from the scarified area. Scarification also removed all woody saplings, although post-fire sapling density did not differ from the undisturbed understory (*H* = 16.05; *df* = 2; *p*<0.001; Fig. 2G).

The disturbed areas had greater slopes than the undisturbed area (*H* = 10.26; *df* = 2; *p* = 0.006; Fig. 2H) although aspect did not differ (*H* = 2.89; *df* = 2; *p* = 0.236; Fig. 2I). Patterns in under-canopy radiation were more similar to basal area than slope (Fig. 2), implying that basal area had a greater influence on radiation.

### Seedling density

**Figure 3:**
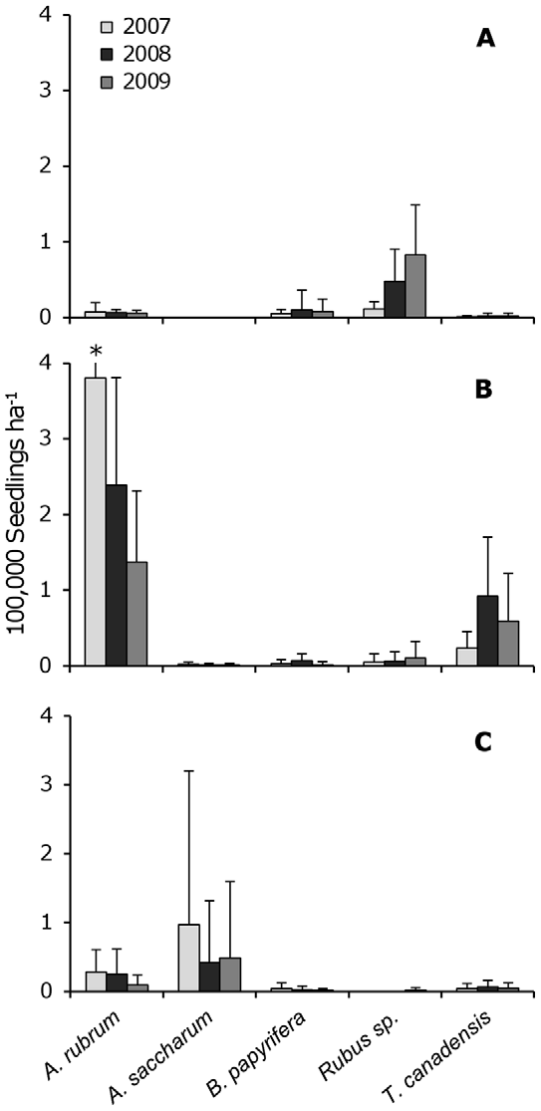
Annual changes in seedling densities by disturbance type. Mean seedling density of the five most abundant woody species by time since disturbance (2007: one year; 2008: two years; 2009: three years) and disturbance type: (**a**) fire, (**b**) scarification, and (**c**) undisturbed. Error bars represent one standard deviation (*n* = 10). Asterisks indicate error bars that exceed the figure scale.

Soil scarification by bulldozer facilitated the greatest eastern hemlock seedling density of the two disturbance types and the undisturbed forest during each year of the study (1 year: *H* = 12.9, *df* = 2; *p* = 0.002; 2 years: *H* = 20.9, *df* = 2; *p*<0.0001; 3 years: *H* = 19.8, *df* = 2; *p*<0.0001; Kruskal-Wallis test results) (Fig. 3). In the scarified area, hemlock seedling density increased from the first to second years after disturbance (*W* = 73.5; *p* = 0.037; Mann-Whitney test results), but did not increase from the second to third years (*W* = 120; *p* = 0.542) (Fig. 3B). In the burned and undisturbed areas, hemlock seedling density remained consistently lower over the three-year study (Fig. 3A, 3C). Hemlock was also a significant indicator species of the scarification disturbance during all three years of the study, based on percent cover (Table 1). In addition to hemlock, red maple was also a significant indicator species of soil scarification (Table 1). Red maple seedling density was greatest in the scarified area throughout the study (1 year: *H* = 21.1, *df* = 2, *p*<0.0001; 2 years: *H* = 21.2, *df* = 2; *p*<0.0001; 3 years: *H* = 19.0, *df* = 2; *p*<0.0001). Compared to hemlock seedling density, red maple was 16 times more abundant in the first year after disturbance and 2.5 times more abundant in the second and third years. Changes in red maple seedling density over time were not statistically significant (1–2 years: *W* = 127; *p* = 0.208, 2–3 years: *W* = 127.5; *p* = 0.192). Northern white-cedar became a significant indicator species of scarification by the third year of the study (Table 1), with a seedling density of 32,667±58,348 seedlings ha^−1^.

**Table 1:** Significant indicator species by disturbance type in 2007; one year after disturbance.

Species	Taxonomic group	Disturbance type	Obs. *IV* a	Rand. *IV* b	*P*
*Pteridium aquilinium*	Cryptogam	Fire	57.1	22.6 (7.78)	0.002
Grasses	Graminoid	Fire	54.9	18.9 (7.61)	0.003
*Rubus* spp.	Shrub	Fire	49.4	25.9 (7.45)	0.013
*Polygonum cilinode*	Forb	Fire	40.6	23.6 (7.57)	0.034
*Acer rubrum*	Tree	Scarification	53.2	34.9 (4.61)	<0.001
*Tsuga Canadensis*	Tree	Scarification	48.6	29.3 (6.48)	0.013

a Observed importance value.

b Mean importance value from randomized groups; standard deviation in parentheses; Monte Carlo test with 10,000 permutations.

In the burned area, paper birch and red maple seedlings were the most abundant trees (Fig. 3A). Both of these species are often associated with post-fire vegetation, especially paper birch. However, paper birch was not a significant indicator of fire even by the third year of the study (*IV* = 15.6; *p* = 0.86). Red maple seedling density remained relatively constant over the three-year study, from 7,000±12,217 seedlings ha^−1^ in the first year to 5,333±3,583 seedlings ha^−1^ in the third year. *Rubus* spp. shrubs composed most of the woody seedling community by the second (47,333±42,855) and third (82,667±66,198) years after disturbance (Fig. 3A). *Rubus* spp. was also a significant indicator of fire each year of the study (Table 1).

Sugar maple and red maple were the most abundant seedlings in the undisturbed reference plots based on seedling density (Fig. 3C), but neither species was a significant indicator based on cover. Sugar maple seedling density was highly variable and decreased by half from the first year (97,000±222,813) to the second year (41,667±89,708) of the study, and leveled off by the third year (48,333±110,680). Red maple seedling density remained similar from the first (28,667±31,903) to the second year (24,667±36,589), but declined by the third year (9,333±14,212). High inter-annual variability in seedling density is common, and may be due to fluctuations in seed production [48]. Both species exhibited a clumped spatial distribution as demonstrated by large standard deviations relative to the mean density, which may explain why sugar maple was not a significant indicator species despite its high mean abundance. There were no indicator species from the undisturbed plots.

### Herbaceous-layer plant communities

The NMDS ordination resulted in a three-axis solution explaining 82% of the variation in the data structure, and with a final stress of 14.2 and an instability <0.001 in 380 iterations. The three axes explained 30%, 20%, and 32% of the variation, respectively. Among the environmental variables, diffuse and direct under-canopy radiation were generally the most correlated with NMDS axes. Axis 1 was most positively correlated with basal area (*τ* = 0.211) and negatively correlated with diffuse under-canopy radiation (*τ* = −0.505), direct under-canopy radiation (*τ* = −0.397), and slope (*τ* = −0.251). Axis 2 was most positively correlated with slope (*τ* = 0.323) and diffuse under-canopy radiation (*τ* = 0.234) and negatively correlated with high decay coarse woody debris (*τ* = −0.212). Similarly to axis 1, axis 3 was most positively correlated with basal area (*τ* = 0.215) and negatively correlated with diffuse under-canopy radiation (*τ* = −0.424) and direct under-canopy radiation (*τ* = −0.295).

Based on the MRPP, plant communities varied significantly by treatment type during each year of the study (1 year: *A* = 0.201, *p*<0.001; 2 years: *A* = 0.289, *p*<0.001; 3 years: *A* = 0.265, *p*<0.001). Pairwise comparisons using Bonferroni-adjusted *p*-values indicated that the disturbance types differed from one another and the control one year (2007) and two years (2008) after the burn, but by year three (2009) plant communities in the scarified area did not differ from those in the undisturbed area (*A* = 0.050; *p* = 0.105). The ISA revealed different dominant species in each disturbance type, with all indicators of the fire being graminoids, forbs, shrubs, and ferns while the scarified area was experiencing significant tree seedling regeneration. In the first year after the burn, four species were more abundant and frequent in the burned area: grasses, the forb *Polygonum cilinode*, the fern *Pteridium aquilinium*, and the shrubs *Rubus* spp. (Table 1). In the second year four additional species became significant indicators of the burned area and remained so in the third year: *Carex* spp., the forbs *Cirsium* spp. and *Epilobium ciliatum*, and the shrub *Vaccinium angustifolium* (Tables 2 and 3). In the scarified area, red maple and eastern hemlock seedlings were the only significant indicators one and two years after disturbance, and northern white-cedar became an indicator in the third year (Tables 1–3). There were no significant indicator species in the undisturbed area during any year (Tables 1–3). Species that were indicators of fire tended to be the most negatively correlated with NMDS axes 1 and 3, which were also negatively correlated with direct and diffuse under-canopy radiation and positively correlated with basal area. Eastern hemlock, an indicator species in the scarified area, was the most positively correlated species with axis 3, which was negatively correlated with under-canopy radiation and positively correlated with basal area.

**Table 2 pone-0043867-t002:** Significant indicator species by disturbance type in 2008; two years after disturbance.

Species	Taxonomic group	Disturbance type	Obs. *IV* a	Rand. *IV* b	*p*
*Polygonum cilinode*	Forb	Fire	78.9	24.1 (7.86)	<0.001
*Rubus* spp.	Shrub	Fire	75.3	26.4 (7.77)	<0.001
Grasses	Graminoid	Fire	67.2	26.6 (8.14)	0.001
*Pteridium aquilinium*	Cryptogam	Fire	57.0	21.3 (7.77)	0.002
*Carex* spp.	Graminoid	Fire	52.5	40.2 (5.94)	0.037
*Cirsium* sp.	Forb	Fire	40.0	14.0 (6.76)	0.022
*Epilobium ciliatum*	Forb	Fire	40.0	14.0 (6.89)	0.025
*Vaccinium angustifolium*	Shrub	Fire	40.0	14.4 (7.09)	0.019
*Tsuga canadensis*	Tree	Scarification	52.7	30.8 (6.56)	0.004
*Acer rubrum*	Tree	Scarification	43.2	36.7 (2.23)	0.002

a Observed importance value.

b Mean importance value from randomized groups; standard deviation in parentheses; Monte Carlo test with 10,000 permutations.

**Table 3:** Significant indicator species by disturbance type in 2009; three years after disturbance.

Species	Taxonomic group	Disturbance type	Obs. *IV* a	Rand. *IV* b	*p*
*Polygonum cilinode*	Forb	Fire	82.3	24.0 (8.00)	<0.001
*Rubus* spp.	Shrub	Fire	78.0	26.6 (8.25)	<0.001
Grasses	Graminoid	Fire	70.5	25.3 (8.08)	0.001
*Epilobium ciliatum*	Forb	Fire	70.0	18.4 (7.39)	0.001
*Carex* spp.	Graminoid	Fire	57.8	40.9 (5.64)	0.007
*Pteridium aquilinium*	Cryptogam	Fire	57.3	23.9 (7.86)	0.003
*Vaccinium angustifolium*	Shrub	Fire	55.5	19.1 (7.97)	0.003
*Cirsium* sp.	Forb	Fire	50.0	15.9 (7.49)	0.007
*Acer rubrum*	Tree	Scarification	55.2	34.2 (5.64)	0.001
*Tsuga canadensis*	Tree	Scarification	54.4	30.5 (6.47)	0.003
*Thuja occidentalis*	Tree	Scarification	34.4	15.5 (7.33)	0.042

a Observed importance value.

b Mean importance value from randomized groups; standard deviation in parentheses; Monte Carlo test with 10,000 permutations.

**Figure 4:**
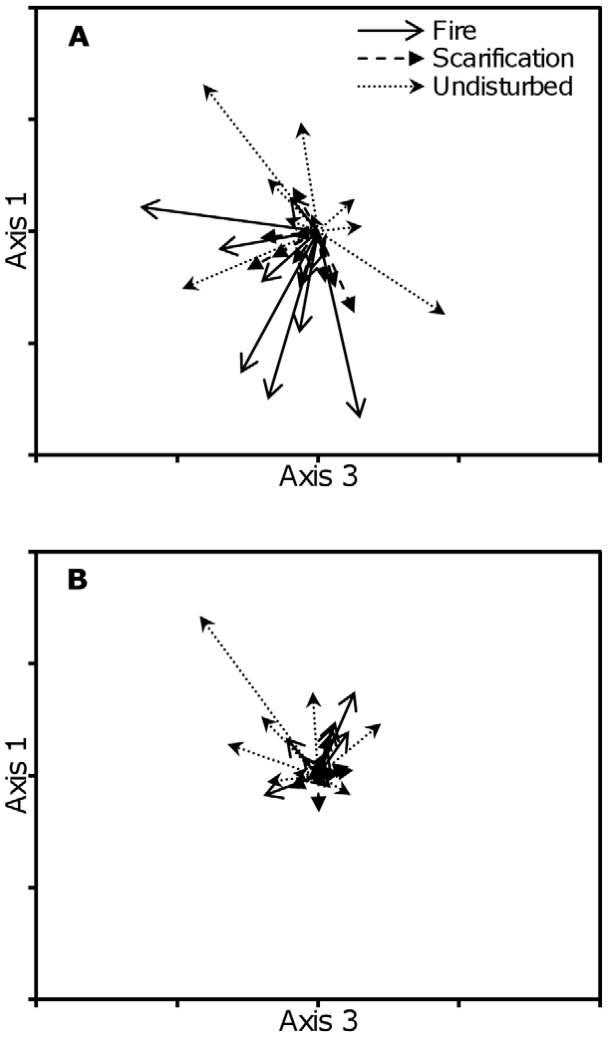
Annual herbaceous-layer community change by disturbance type. Non-metric multidimensional scaling (NMDS) ordination scores depicting relative changes in species composition by disturbance type and years since disturbance. Each vector represents the magnitude and direction of change for each plot from one to two years (**a**; 2007–2008) and two to three years (**b**; 2008–2009) after disturbance. The NMDS ordination was three-dimensional with a final stress 14.2 and instability <0.001 in 380 iterations. Only the two dominant axes are displayed (Axis 3: *r^2^* = 0.32; Axis 1: *r^2^* = 0.30) whereas the third axis (Axis 2: *r^2^* = 0.20) is excluded.

Species composition changed more rapidly in the burned area than the scarified or undisturbed areas in the first two years after disturbance, but this change decreased by the third year. The MRPP of standardized NMDS scores revealed significant differences between treatments in species change from one to two years (*A* = 0.099; *p*<0.001) and from two to three years (*A* = 0.056; *p* = 0.012) after disturbance. However, an examination of trends in pairwise comparisons and vector plots (Fig. 4) suggests that the significant result from years two to three is due in large part to an outlier in the reference area. Based on pairwise comparisons of Bonferroni-adjusted *p*-values, the change in community composition from years one to two was greater in the burned area than the scarified (*A* = 0.090; *p* = 0.009) or undisturbed (*A* = 0.089; *p* = 0.012) area, while the scarified and undisturbed area did not differ from one another (*A* = 0.048; *p* = 0.135). From years two to three, the burned area did not differ from the scarified area (*A* = 0.007; *p* = 0.888) or the undisturbed area (*A* = 0.028; *p* = 0.283), but the scarified and undisturbed areas were significantly different (*A* = 0.083; *p* = 0.010). Due to the single outlier in the reference area and the relatively low *A*, we conclude that differences in NMDS vectors from years two to three are not likely ecologically significant even if they are significant statistically.

## Discussion

Eastern hemlock regeneration was more likely to occur following an understory disturbance that exposed mineral soil and reduced herbaceous-layer competition compared to either canopy removal by fire or a lack of disturbance. In our study, soil scarification by a bulldozer increased exposure of the mineral soil and eliminated competition from the sapling layer without substantially altering understory light levels. This combination of environmental factors allowed hemlock seedlings to establish more readily compared to burning and lack of disturbance. Red maple seedlings established rapidly after scarification, but were decreasing in abundance by the third year after disturbance. Paper birch seedlings were not among the most abundant species in the burned area as we expected. Herbaceous and shrub species that are often associated with crown fire and other canopy disturbances increased in cover during the 3-year study period. In the adjacent undisturbed area, no herbaceous-layer species was consistently abundant enough to be considered a significant indicator. Although we recorded several species commonly associated with hemlock forest, including *Maianthemum canadense*, *Trientalis borealis*, *Dryopteris* sp., *Oxalis* sp., *Coptis trifolia*, *Lycopodium* spp., and *Carex* spp. [49], they were present in low abundance and frequency (Table S1).

Our results corroborate and augment previous silvicultural recommendations for hemlock, based on hemlock life history and case studies. Hemlock is a long-lived (up to 800 years), slow-growing (up to 250–300 years to reach maturity), and highly shade-tolerant conifer [49]. A mature tree produces copious amounts of seed annually, but successful seedling establishment is rare [49]. Soil conditions that increase the success of establishment are warm temperatures (7°–18°C), high moisture content [49], and a suitable germination substrate of exposed mineral soil or coarse woody debris [28], [50], [51]. Hemlock seedlings rarely germinate through thick, dense litter layers [28], [51]. Natural hemlock regeneration is increased by single treefall events that disturb the soil and create a small canopy opening [15], [26]. Silvicultural recommendations for germinating hemlock include soil preparations of mixing the organic and upper mineral horizons or burning with a surface fire [49]. Greater seedling densities have been observed where the soil was mechanically scarified by a bulldozer and rock rake [52], and following a prescribed burn [7]. In a greenhouse experiment using excavated soil monoliths, seedling emergence was greatest when the organic layer was removed compared to burning or retaining the organic layer [53].

**Figure 5:**
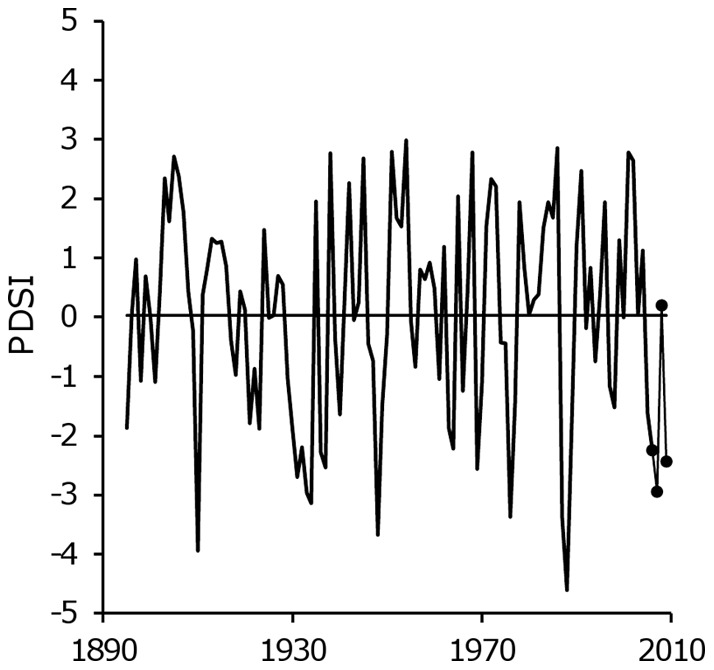
Drought conditions occurred in northern Wisconsin during 2007 and 2009. Growing-season (May-September) Palmer Drought Severity Index (PDSI) for Wisconsin Climate Division 1 is plotted by year (National Climatic Data Center, 2012). PDSI values during the sampling period are indicated by dark circles, and the 115-year mean is plotted as a straight line.

Although hemlock seedling establishment occurred in the scarified area, successful hemlock regeneration may be tempered by drought. Mladenoff and Stearns [26] hypothesized that successful hemlock regeneration after a disturbance event depends on climatic conditions in the decades after disturbance. Paleoecological studies have indicated that hemlock's range expanded and contracted with changes in temperature and precipitation in the northern United States and southern Canada during the Holocene epoch [54], [55]. For example, prior to 3500–3000 years before present, sites currently occupied by hemlock in the northern Lake States were likely dominated by white pine [4], [5]. The transition to hemlock dominance corresponded with increased moisture availability and decreased fire frequency across the region. During our 3-year study, northern Wisconsin experienced growing-season drought conditions as demonstrated by the Palmer Drought Severity Index (PDSI; [56]) during all but the second year (2008), which was near average (Fig. 5). The second year corresponded with an increase in hemlock seedling density as well as northern white-cedar, another species whose seedlings are sensitive to desiccation [57]. Seedling density of these species did not continue to increase in the third year when drought conditions returned. Although our study occurred over a short time scale of 3 growing seasons, our results corroborate other studies that suggest hemlock's range may contract if drier growing-season conditions accompany warming temperatures [58]. Recent climate models predict increased growing season temperatures in the northern Great Lakes region over the next century, with high uncertainty for precipitation [59]. If consistently moist growing season conditions become less frequent in hemlock-hardwood forests, then species whose seedlings are less susceptible to desiccation are likely to capture disturbed sites at the expense of hemlock.

Red maple is one species that could potentially increase in response to drought and disturbance in hemlock-hardwood forests, at least in the immediate future. Red maple is a habitat generalist that has thrived in North America following European settlement, due in part to increased rates of forest disturbance [60]. Hibbs [61] reported that red maple seedlings grew faster than hemlock seedlings in hemlock-hardwood forest canopy gaps. Although red maple often fails to dominate the canopy due to decreasing shade tolerance with tree size, Walters and Yawney [62] report cases of overstory red maple density increasing following disturbance. In the current study, red maple seedlings established at high densities in the scarified plots even during the drought year of 2007. Red maple seedling density was similar to that of paper birch in the burned area, suggesting that red maple trees may eventually be present in the burned area as well. An important caveat of our hypothesis is that areas with high white-tailed deer populations may not see an increase in red maple because it is a preferred browse species [33]. We did not observe a great amount of browse damage at our study site, and the seedlings we censused were not yet tall enough to be browsed by deer.

Forest herbaceous-layer vegetation functions as a filter for overstory regeneration. Initial post-disturbance vegetation communities greatly affect both the future overstory composition and rate of succession [35]. In the burned area of our study, the establishment of low-stature ferns, shrubs, and graminoids may be slowing the establishment of tree seedlings. George and Bazzaz [63] observed significantly reduced seedling germination and establishment of sweet birch (*Betula lenta*) and yellow birch in the presence of ferns, whereas red maple seedlings were not negatively impacted by ferns. *Rubus* spp. is known to rapidly increase in cover in post-fire forest understories, reducing the rate of seedling establishment [64]. In the burned area of our study, it is conceivable that *Rubus* spp. suppressed tree seedling establishment given that it was 11 times more abundant than the most abundant tree seedling paper birch. However, *Rubus* spp. may eventually facilitate tree regeneration by protecting seedlings from desiccation and increasing nitrogen availability [64]. At three years after fire, it is unclear which tree species will achieve canopy dominance in our study site, but rapid increases in herbaceous and shrub densities are likely slowing the rate of succession.

## Conclusions

Our results suggest that soil scarification is a useful method of regenerating eastern hemlock in hemlock-hardwood forests, but the climatic conditions that follow soil disturbance may modulate regeneration success. Although fire is not facilitating immediate hemlock re-establishment in the current study, several cases of fire-origin hemlock stands have been observed. Although we cannot test this hypothesis with our current data, we suggest that these stands may have regenerated when wetter climate conditions followed a fire. Therefore, hemlock and other late-successional temperate tree species may be difficult to regenerate, especially if the climate in temperate regions becomes warmer as predicted. Where hemlock regeneration is desired, planting of saplings and/or removal of competitors may help to circumvent the potential consequences of drought.

Forest management actions that focus on foundation species such as hemlock are more likely to achieve broad success by preserving the ecological interactions associated with the focal species. In unmanaged forest of the upper Great Lakes region, current rates of hemlock regeneration are insufficient to maintain regional hemlock dominance [24], [33], although regeneration may be abundant in select locations [28]. Past extirpations of foundation species, such as American chestnut (*Castanea dentata*), have led to widespread and unpredictable ecosystem change [20]. Therefore, appropriate management of existing foundation species is important to conservation of forest ecosystem function.

## Supporting Information

Table S1 **Mean percent cover of herbaceous layer vegetation (<50 cm height) in each treatment type (F =  Fire, S =  Scarification, U =  Undisturbed) recorded one year (2007), two years (2008), and three years (2009) after disturbance.**
(DOCX)
