# Supplementary material for: Post-Disturbance Plant Community Dynamics following a Rare Natural-Origin Fire in a Tsuga canadensis Forest
Source: PLoS One. 2012 Aug 21;7(8):e43867. doi: 10.1371/journal.pone.0043867 (PMC3424231; doi:10.1371/journal.pone.0043867)
Supplement: Table S1 — Mean percent cover of herbaceous layer vegetation (<50 cm height) in each treatment type (F = Fire, S = Scarification, U = Undisturbed) recorded one year (2007), two years (2008), and three years (2009) after disturbance. (DOCX) [file pone.0043867.s001.docx]

| **Species** | | | **Authority** | | | **Tax. group** | | **F-07** | | **S-07** | | **R-07** | | **F-08** | | **S-08** | | **R-08** | | **F-09** | | **S-09** | | **R-09** | |
| --- | --- | --- | --- | --- | --- | --- | --- | --- | --- | --- | --- | --- | --- | --- | --- | --- | --- | --- | --- | --- | --- | --- | --- | --- | --- |
| *Abies balsamea* | | | (L.) P. Mill. | | | Tree | | T^a^ | | -^b^ | | 0.1 | | T | | 0.1 | | 0.1 | | T | | T | | 0.1 | |
| *Acer rubrum* | | | L. | | | Tree | | 0.2 | | 0.9 | | 0.3 | | 0.3 | | 0.8 | | 0.4 | | 0.3 | | 1.1 | | 0.4 | |
| *Acer saccharum* | | | Marsh. | | | Tree | | - | | 0.1 | | 4.1 | | - | | 0.1 | | 2.8 | | - | | 0.1 | | 1.5 | |
| *Acer spicatum* | | | Lam. | | | Tree | | - | | - | | T | | - | | - | | 0.1 | | - | | - | | 0.1 | |
| *Amelanchier* sp. | | | Medik. | | | Tree | | - | | - | | - | | - | | - | | T | | - | | - | | T | |
| *Aralia hispida* | | | Vent. | | | Forb | | - | | - | | - | | 0.1 | | T | | - | | 0.4 | | 0.1 | | - | |
| *Aralia nudicaulis* | | | L. | | | Forb | | T | | 0.1 | | 0.2 | | T | | T | | - | | 0.2 | | T | | T | |
| *Arctostaphylos uva-ursi* | | | L. | | | Shrub | | 0.1 | | - | | - | | 0.2 | | - | | - | | 0.4 | | - | | - | |
| *Betula alleghaniensis* | | | Britt. | | | Tree | | - | | - | | T | | 0.1 | | 0.1 | | - | | T | | 0.1 | | T | |
| *Betula papyrifera* | | | Marsh. | | | Tree | | 0.2 | | 0.1 | | 0.1 | | 0.1 | | 0.1 | | 0.1 | | 0.1 | | 0.1 | | 0.1 | |
| *Carex* spp. | | | L. | | | Graminoid | | 5.3 | | 1.1 | | 1.5 | | 14.5 | | 4.4 | | 1.3 | | 16.0 | | 5.9 | | 1.0 | |
| *Chamerion* sp. | | | Raf. ex Holub | | | Forb | | - | | - | | - | | T | | - | | - | | 0.1 | | - | | - | |
| *Circaea lutetiana* | | | L. | | | Forb | | - | | T | | - | | - | | - | | - | | - | | - | | - | |
| *Cirsium* sp. | | | P. Mill. | | | Forb | | - | | - | | - | | 0.1 | | - | | - | | 0.3 | | - | | - | |
| *Clematis* sp. | | | L. | | | Forb | | - | | - | | 0.1 | | - | | - | | T | | - | | - | | T | |
| *Clintonia borealis* | | | (Ait.) Raf. | | | Forb | | T | | - | | - | | 0.1 | | T | | - | | 0.2 | | T | | - | |
| *Comptonia peregrina* | | | (L.) Coult. | | | Shrub | | 0.1 | | - | | - | | 0.1 | | - | | - | | 0.1 | | - | | - | |
| *Coptis trifolia* | | | (L.) Salisb. | | | Forb | | 0.1 | | T | | 0.1 | | 0.1 | | 0.1 | | 0.1 | | 0.1 | | 0.1 | | 0.1 | |
| *Cornus canadensis* | | | L. | | | Forb | | 0.2 | | 0.1 | | 0.4 | | 0.6 | | T | | 0.3 | | 1.7 | | 0.1 | | 0.5 | |
| *Corydalis sempervirens* | | | (L.) Pers. | | | Forb | | - | | 0.1 | | - | | - | | - | | - | | - | | - | | - | |
| *Corylus cornuta* | | | Marsh. | | | Shrub | | 0.1 | | - | | 0.2 | | 0.1 | | T | | 0.3 | | 0.3 | | 0.1 | | 0.3 | |
| *Diervilla lonicera* | | | P. Mill. | | | Shrub | | - | | 0.1 | | - | | T | | 0.4 | | - | | 0.5 | | 0.8 | | - | |
| *Dryopteris* sp. | | | Adans. | | | Cryptogam | | - | | - | | 1.5 | | - | | - | | 1.0 | | - | | - | | 1.1 | |
| *Epilobium ciliatum* | | | Raf. | | | Forb | | - | | - | | - | | 0.1 | | - | | - | | 0.2 | | - | | - | |
| *Equisetum arvense* | | | L. | | | Cryptogam | | - | | - | | - | | 0.1 | | - | | - | | T | | - | | - | |
| *Equisetum sylvaticum* | | | L. | | | Cryptogam | | - | | - | | - | | - | | - | | - | | 0.1 | | - | | - | |
| *Euthamia graminifolia* | | | (L.) Greene | | | Forb | | - | | - | | - | | - | | - | | - | | 0.1 | | - | | - | |
| *Fraxinus nigra* | | | Marsh. | | | Tree | | - | | - | | T | | - | | - | | T | | - | | - | | T | |
| *Galeopsis tetrahit* | | | L. | | | Forb | | 0.1 | | - | | T | | 0.1 | | - | | T | | 0.1 | | - | | - | |
| *Galium triflorum* | | | Michx. | | | Forb | | T | | 0.1 | | - | | 0.4 | | 0.5 | | - | | 0.8 | | 0.5 | | - | |
| *Gaultheria procumbens* | | | L. | | | Shrub | | T | | T | | T | | 0.1 | | T | | - | | 0.1 | | T | | - | |
| Grass | | |  | | | Graminoid | | 0.4 | | T | | - | | 1.8 | | 0.1 | | - | | 2.1 | | 0.1 | | T | |
| *Hepatica nobilis* | | | Schreber | | | Forb | | - | | - | | T | | - | | - | | T | | - | | - | | 0.1 | |
| *Hieracium* sp. | | | L. | | | Forb | | - | | - | | - | | - | | - | | - | | 0.1 | | - | | - | |
| *Huperzia lucidula* | | | Michx. | | | Cryptogam | | - | | - | | T | | - | | - | | 0.1 | | - | | - | | T | |
| *Hydrocotyle americana* | | | L. | | | Forb | | - | | - | | - | | - | | - | | T | | - | | - | | T | |
| *Leucanthemum vulgare* | | | Lam. | | | Forb | | - | | - | | - | | T | | - | | - | | 0.1 | | - | | - | |
| *Lonicera canadensis* | | | Bartr. ex Marsh. | | | Shrub | | T | | 0.2 | | T | | T | | 0.1 | | T | | - | | 0.1 | | T | |
| *Lycopodium annotinum* | | | L. | | | Cryptogam | | - | | - | | 0.5 | | T | | - | | 0.6 | | T | | - | | 0.5 | |
| *Lycopodium dendroideum* | | | Michx. | | | Cryptogam | | 0.3 | | - | | 0.9 | | 0.3 | | 0.1 | | 0.6 | | 0.3 | | T | | 0.6 | |
| *Maianthemum canadense* | | | Desf. | | | Forb | | 0.4 | | 0.5 | | 0.5 | | 1.7 | | 1.2 | | 0.6 | | 2.5 | | 1.8 | | 0.5 | |
| *Maianthemum racemosum* | | | (L.) Link | | | Forb | | - | | T | | - | | - | | - | | - | | - | | - | | - | |
| *Mitchella repens* | | | L. | | | Forb | | T | | T | | T | | - | | T | | - | | - | | T | | T | |
| Moss | | |  | | | Cryptogam | | 0.8 | | 0.2 | | 5.5 | | 0.9 | | 1.0 | | 6.3 | | 1.3 | | 1.9 | | 4.6 | |
| *Ostrya virginiana* | | | (P. Mill.) K. Koch | | | Tree | | - | | T | | - | | - | | - | | - | | - | | - | | T | |
| *Oxalis* sp. | | | L. | | | Forb | | - | | - | | T | | - | | - | | T | | - | | - | | T | |
| *Picea glauca* | | | (Moench) Voss | | | Tree | | - | | - | | - | | - | | 0.1 | | - | | T | | T | | - | |
| *Pinus strobus* | | | L. | | | Tree | | - | | 0.1 | | - | | - | | - | | - | | - | | - | | - | |
| *Polygala paucifolia* | | | Willd. | | | Forb | | - | | - | | - | | 0.1 | | - | | - | | T | | - | | - | |
| *Polygonatum pubescens* | | | (Willd.) Pursh | | | Forb | | 0.1 | | T | | 0.1 | | T | | T | | 0.3 | | 0.1 | | T | | 0.3 | |
| *Polygonum cilinode* | | | Michx. | | | Forb | | 0.7 | | 0.2 | | 0.1 | | 5.5 | | 0.4 | | - | | 7.8 | | 0.3 | | - | |
| *Populus grandidentata* | | | Michx. | | | Tree | | - | | - | | - | | T | | - | | - | | T | | - | | - | |
| *Populus tremuloides* | | | Michx. | | | Tree | | - | | - | | - | | - | | - | | - | | T | | - | | - | |
| *Prunus pensylvanica* | | | L. f. | | | Tree | | 0.4 | | 0.2 | | - | | 0.4 | | 0.2 | | - | | 0.1 | | 0.2 | | - | |
| *Prunus virginiana* | | | L. | | | Tree | | - | | - | | - | | T | | - | | - | | 0.1 | | - | | - | |
| *Pteridium aquilinium* | | | (L.) Kuhn | | | Cryptogam | | 2.7 | | 0.3 | | 0.1 | | 1.1 | | 0.2 | | T | | 1.5 | | 0.3 | | 0.2 | |
| *Quercus rubra* | | | L. | | | Tree | | T | | - | | - | | T | | - | | - | | T | | - | | - | |
| *Ribes* sp. | | | L. | | | Shrub | | - | | T | | - | | T | | - | | - | | T | | - | | - | |
| *Rubus* spp. | | | L. | | | Shrub | | 0.5 | | 0.4 | | - | | 3.3 | | 0.2 | | 0.1 | | 7.5 | | 0.4 | | T | |
| *Sambucus racemosa* | | | L. | | | Shrub | | - | | - | | - | | 0.1 | | - | | - | | T | | - | | - | |
| *Silene vulgaris* | | | (Moench) Garcke | | | Forb | | - | | 0.1 | | - | | - | | 0.1 | | - | | - | | 0.1 | | - | |
| *Solidago* sp. | | | L. | | | Forb | | - | | - | | - | | T | | - | | - | | 0.1 | | - | | - | |
| *Streptopus lanceolatus* | | | (Ait.) Reveal | | | Forb | | - | | T | | T | | - | | T | | T | | - | | - | | - | |
| *Taraxacum officinale* | | | G.H. Weber ex Wiggers | | | Forb | | - | | - | | - | | T | | T | | - | | T | | T | | - | |
| *Thuja occidentalis* | | | L. | | | Tree | | - | | - | | - | | - | | 0.2 | | 2.4 | | - | | 0.2 | | T | |
| *Toxicodendron radicans* | | | (L.) Kuntze | | | Forb | | - | | T | | - | | - | | - | | - | | - | | - | | - | |
| *Trientalis borealis* | | | Raf. | | | Forb | | T | | - | | 0.2 | | 0.1 | | 0.1 | | 0.2 | | 0.1 | | T | | 0.3 | |
| *Tsuga canadensis* | | (L.) Carr. | | | Tree | | | T | | 0.3 | | 0.2 | | 0.1 | | 0.4 | | 0.5 | | 0.2 | | 0.4 | | 0.3 | |
| Unknown aster | | L. | | | Forb | | | - | | - | | T | | T | | - | | T | | - | | - | | T | |
| Unknown forb | |  | | | Forb | | | - | | - | | - | | - | | - | | - | | 0.4 | | 0.5 | | 0.1 | |
| *Vaccinium angustifolium* | | Ait. | | | Shrub | | | 0.3 | | - | | T | | 0.4 | | - | | - | | 0.6 | | - | | T | |
| *Verbascum* sp. | | L. | | | Forb | | | - | | - | | - | | - | | - | | - | | T | | - | | - | |
| *Veronica officinalis* | | L. | | | Forb | | | - | | - | | - | | - | | T | | - | | - | | - | | - | |
| *Viola* spp. | | L. | | | Forb | | | - | | 0.1 | | - | | 0.1 | | 0.1 | | - | | 0.2 | | 0.1 | | - | |

^a^ *T* trace amounts (< 0.1%)

^b^ - absent
